# Latest Trends in Hemodiafiltration

**DOI:** 10.3390/jcm13041110

**Published:** 2024-02-16

**Authors:** Francisco Maduell, Diana Rodríguez-Espinosa, José Jesús Broseta

**Affiliations:** Department of Nephrology, Hospital Clínic, 08036 Barcelona, Spain; dmrodriguez@clinic.cat (D.R.-E.); jjbroseta@clinic.cat (J.J.B.)

**Keywords:** hemodiafiltration, dialyzer compatibility, survival, safety

## Abstract

This review provides a detailed analysis of hemodiafiltration (HDF), its progress from an emerging technique to a potential conventional treatment for chronic hemodialysis patients, and its current status. The article covers the advances, methods, and clinical benefits of HDF, specifically focusing on its impact on cardiovascular health, survival rates, and overall well-being. The review also addresses questions about the safety of HDF and provides evidence to dispel concerns related to the elimination of beneficial substances and infection risks. Additionally, the article explores the potential implications of expanded hemodialysis (HDx) as an alternative to HDF, its classification, safety profile, and an ongoing trial assessing its non-inferiority to HDF. Supported by evidence from randomized controlled trials and observational studies, the review emphasizes the superiority of HDF as a hemodialysis modality and advocates for its positioning as the gold standard in treatment. However, it acknowledges the need for extensive research to define the role of HDx in comprehensive treatment approaches in individuals undergoing dialysis. The synthesis of current knowledge underscores the importance of ongoing exploration and research to refine hemodialysis practices for optimal patient outcomes.

## 1. Introduction

After hemodiafiltration (HDF) was first introduced by Lee Henderson in 1973 in the USA [1] and on-line substitution fluid generation was in practice in the mid-1980s in Europe, HDF has evolved significantly [2,3]. This review explores its trajectory from an emerging technique to a potentially conventional treatment for chronic hemodialysis patients. The article provides in-depth discussion of the advancements, method, dialyzer compatibility, clinical advantages, current evidence, ongoing research, and the potential implications of expanded hemodialysis (HDx) as an alternative modality. Although HDF was introduced in Europe in the mid-1980s, in 2015, the American National Kidney Foundation clinical practice guideline for hemodialysis (HD) adequacy devoted only a single paragraph to this modality [4]. This working group acknowledged that HDF was not widely available in the US. In 2018, more than 20 years after its introduction in Europe, the US formally stated regulatory considerations for HDF and asked whether it addresses unmet medical needs. In this review, we discuss whether HDF can currently be considered as a conventional treatment for HD patients.

## 2. Evolution of Hemodialysis

During the 1980s, conventional HD involved dialysis using acetate dialysate, dialysis machines that did not have volumetric control, low blood flow, and low-flux dialyzers [5]. However, in the 1990s, the concept of conventional HD changed due to technological advances in dialysis machines, ultrafiltration control, and the widespread use of bicarbonate dialysate. This allowed for an increase in blood flow and the use of synthetic high-flux dialyzers [5]. To avoid adverse reactions caused by backfiltration, exogenous replacement fluid was promoted. However, it was limited due to technical and financial reasons [6].

This scenario changed in the mid-1980s with the development of online HDF techniques using the dialysate as a replacement fluid. The concept of conventional HD has continuously evolved over the years and largely depends on the possibilities and technological availability in each dialysis unit. Over the past twenty years, there have been significant advancements in high-volume hemodiafiltration (HDF) techniques. The question now is whether HDF can be considered a conventional treatment for patients with chronic hemodialysis. High-convection-volume HDF techniques constitute progress towards renal replacement therapy, which is most similar to the natural function of the kidney. This technique is indicated in all patients because there are no contraindications [5].

## 3. Characteristics of Hemodiafiltration

In 2013, the European convective working group [7] defined HDF as hemodialysis with a high-flux dialyzer and ultrafiltration coefficient greater than 20 mL/mmHg/h/m^2^, sieving coefficient ß_2_-microglobulin > 0.6 and effective convection volume of at least 20% of the total blood processed. 

The convective volume comprises the substitution volume and the ultrafiltered interdialysis weight gain during the dialysis session [8]. The substitution volume can be delivered to the patient, either as predilution (prefilter) or postdilution (postfilter) [9]. 

The EuDial group specified that the effective convection volume is the total volume of undiluted ultrafiltered fluid. Therefore, it is necessary to calculate the dilution factor. Moreover, the group suggested that infusion flow should be at least twice the postdilution infusion for an equal clearance in predilution. We know that predilution infusion can slightly decrease the clearance of small molecules, while the clearance of large molecules is achieved by the convective volume, but always somewhat lower than postdilution. This is why the most widely used method is postdilution HDF in Europe, except in Japan, where the use of predilutional HDF is usually favored [7].

Blood flow is an important factor in achieving an adequate convective volume. Modern dialysis machines deliver the substitution volume automatically, influenced mainly by the prescribed Qb. For instance, the substitution volume increased from 23 L to 35 L when the Qb increased from 250 mL/min to 450 mL/min. An interesting observation is how the auto substitution algorithm reaches 33% of the filtration fraction with a Qb of 250 mL/min, while it decreases progressively to 27% with a Qb of 450 mL/min [10].

## 4. Dialyzer Compatibility and Safety

In the year 2000, a study by Maduell et al. concluded that most high-flux dialyzers were suitable for HDF except for two, symmetric cellulose triacetate (CTA) and polymethylmethacrylate (PMMA) dialyzers, which should be limited to high-flux hemodialysis (HF-HD), given their association with elevated transmembrane pressure [11].

The former has been demonstrated to be useful in hypersensitivity reactions to the use of synthetic HD membranes. These disappear when patients are switched to a CTA dialyzer, making this the main reason for its prescription [12]. It has been estimated that around 2% of dialysis patients have had a hypersensitivity reaction and require the use of a CTA dialyzer. In this context, the industry developed a new generation of CTA. The main modifications were a change from a homogeneous to an asymmetric structure and a decrease in its roughness. This immediately begged the question of whether this new series of dialyzers would be adequate to perform HDF, unlike previous generations [13]. The new asymmetric cellulose triacetate dialyzer increased the ß_2_-microglobulin reduction ratio from 71% in HD to 80% with HDF, myoglobin from 70% to 82%, and prolactin from 61% to 74%. On the other hand, the previous generation of CTA, despite achieving 20 L of convective volume, underperformed, showing a decrease in the reduction ratios of ß_2_-microglobulin, myoglobin, and prolactin in HDF mode compared to HD. In both dialyzers, dialysate albumin losses were around 1 g in HD and 1.7 g in HDF. This new asymmetric CTA generation dialyzer outperformed its predecessor, which indicated that could be used for both the HD modality and postdilution HDF. The previous-generation CTA prescription should be limited to HD [14].

The second dialyzer unsuitable for HDF was PMMA, a membrane with high adsorption properties. Again, the industry has evolved and created a new generation of PMMA, the NF series, designed to suppress platelet adhesion on the membrane. This series has adsorption properties similar to conventional PMMA [15]. When these were compared with polysulfone/helixone in HD mode and postdilution HDF, the only difference was the convective volume, which was 29 L with PMMA and 34 L with polysulfone or helixone. However, the efficacy of the PMMA NF series in HDF was higher than that in HD treatment, and the albumin dialysate loss was less than 1 g with the NF series, which was significantly lower than with either polysulfone or helixone dialyzers [16].

Another debate regarding dialyzers is whether increasing the internal diameter generates a greater replacement volume and enhances efficacy. A study comparing dialyzers with an internal diameter of 185 microns with those with internal diameters of 210 microns found no differences in the convective volume achieved, 32 L with the 1.4 m^2^ dialyzers and 34 L with the 1.8 m^2^ dialyzers, while maintaining a similar efficacy in solute-clearance [17].

## 5. Clinical Advantages of HDF

HDF has demonstrated a spectrum of clinical benefits that strongly impact the cardiovascular health and overall well-being of patients undergoing this treatment. HDF both reduces cardiovascular risk and enhances survival rates among individuals. Notably, it provides superior control over several critical health parameters: it effectively manages hyperphosphatemia [18], improves inflammatory status [19], and optimizes erythropoietin response for better anemia management [20,21]. Moreover, HDF ensures enhanced hemodynamic stability [22,23] and superior control over fluid overload [24], left ventricular hypertrophy [25], and arterial endothelial function [26], thereby reducing the propensity for serum calcification [27]. Notably, the occurrence of neurological symptoms such as restless leg syndrome, polyneuropathy, and itching, which often arise due to the accumulation of medium to large-sized molecules, is substantially reduced [28]. HDF also helps to ameliorate joint pain and dialysis-related amyloidosis, thereby enhancing overall quality of life and satisfaction among patients [29]. Furthermore, the treatment significantly reduces levels of DNA damage [30] and improves antioxidant status, underscoring its multifaceted positive impact on patient health and well-being [31].

The safety of HDF has also been demonstrated. The fear that HDF could eliminate substances beneficial to patients, including vitamins and amino acids, has not been substantiated to date. There have been no case reports or published series of contamination or infection of patients in a dialysis unit due to the use of this treatment. What seems striking is that, after more than 25 years of the clinical application of HDF, no negative studies have been published [5].

## 6. Current Evidence and Ongoing Research

Observational studies have demonstrated numerous clinical benefits and enhanced survival among patients undergoing HDF compared to HF-HD. These benefits range from 22% to 50%, but the need for prospective randomized studies to validate these findings was evident [32,33,34,35,36].

Between 2010 and 2017, five randomized controlled trial (RCTs) were conducted, including the CONTRAST study [37], the Turkish study [38], and the ESHOL study [39], with mortality as the primary endpoint (Table 1). In the CONTRAST and Turkish studies, no survival differences were observed between the groups at the end of the study after an average follow-up of 3 years. However, the ESHOL study demonstrated the superiority of HDF over high-flux HD (hazard ratio, 0.70 [0.53 to 0.92]). The main difference between the ESHOL study and its predecessors was the convective volume achieved, which was much larger in the former, with a median value of 23 L per session. In addition, a sub-analysis of the Turkish study found that survival was longer in patients with a median convective volume greater than 17.4 L than in those with lower volumes. A reanalysis of the ESHOL study that incorporated patients who discontinued treatment and applied an intent-to-treat (ITT) approach revealed a 24% lower all-cause mortality in the HDF arm, compared to 30% when these patients were censored. Similarly, renal transplantation, as a competing event, produced consistent results.

The EuDial Pooling project analysis indicated significant reductions in all-cause and cardiovascular mortality by 14% and 33%, respectively, in the HDF arm [41]. These findings were corroborated by meta-analyses that supported the beneficial effects of HDF over HD in reducing mortality rates. In recent years, different observational studies and registries from different countries have been published, which, although they do not have the same scientific evidence as the RCT due to their indication and inclusion bias, they all agree that patients in the HDF arm present better results. National registries from various countries, including France [42], Australia, New Zealand [43], Argentina [44], and Brazil [45], consistently demonstrated lower all-cause and cardiovascular mortality rates among patients undergoing HDF than those on HD. The French registry analyzed more than 2,000 patients on HDF exclusively and found a hazard ratio of 0.77 (95% CI, 0.67–0.87) and 0.66 (95% CI, 0.50–0.86) for all-cause and cardiovascular mortality, respectively [42]. The Australia and New Zealand registry analyzed data from around 4,000 patients on HDF and also found a benefit in terms of all-cause mortality with HDF over conventional HF-HD with an adjusted hazard ratio of 0.79 (95% CI, 0.72–0.87) in Australia and 0.88 (95% CI, 0.78–1.00) in New Zealand [43]. Finally, the Argentinian registry also found a 53% reduction in mortality rates in patients on HDF compared to those on HF-HD [44].

Observational studies from Colombia [46] and Russia [47] further highlighted the advantages of HDF in reducing mortality. The Colombian study found a statistically significant reduction of 55% in all-cause mortality but not in cardiovascular mortality [46]. The Russian study reported better 5-year survival among patients on HDF with a substitution volume higher than 23–25 L [47]. In addition, a study from Bulgaria reported enhanced quality of life, fewer annual hospitalizations, a lower incidence of cardiovascular events, less itching, and improved hypertension control, chronic joint pain, and nutritional status in patients on HDF.

Various studies underscored the association of high convective volumes in HDF with improved survival rates. The current recommendations suggest a minimum replacement volume in postdilution HDF to ensure optimal clinical benefits [48,49].

In 2023, the CONVINCE trial [40] replicated similar results to those of ESHOL published 10 years previously [39] (Table 2), finding a significant 23% reduction in all-cause mortality, mostly driven by a decrease in infection-related mortality. A subgroup analysis showed that older patients, non-diabetics, those with a fistula as their vascular access, and those without a history of cardiovascular disease seemed to derive the greatest benefit. The lower overall risk of death found in the CONVINCE trial (7.13%) than reported in the European Renal Association registry [50] can be partly attributed to selection bias by enrolling patients who were likely to reach the highest convection volumes [40]. Consequently, the number needed to treat (NNT) was higher in the CONVINCE trial than in ESHOL (22 vs. 10, respectively) [40,51].

Currently, H4RT is an ongoing British RCT [52] that aims to definitively confirm the superiority of high-volume HDF versus HD not only clinically but also in terms of cost-effectiveness. The comparative efficacy between HDF and HD should be sufficiently conclusive, although many specialists remain skeptical due to the cost of the treatment and the apparent lack of benefit in certain subpopulations. The H4RT trial will offer a more comprehensive understanding of the advantages and disadvantages of high-dose HDF compared to high-flux HD in financial terms, thereby shaping treatment approaches for patients undergoing dialysis. There is still room for further study to determine which patient groups might derive the most benefit from HDF treatment.

It is important to take into account that not all studies have reached the same conclusions about the effectiveness of HDF. Although most studies have reported positive results, some have been inconclusive. For instance, the DOPPS study published in 2018 [53] did not support the notion that HDF is superior to HD regarding patient survival. This contradicts the findings of the DOPPS study published in 2006 [35]. Similarly, a meta-analysis of convective techniques [54], which included a study that weighted 20% of it in which acetate-free biofiltration treatment with less than 8 L bicarbonate replacement volume per session was used, concluded that convective dialysis might reduce cardiovascular mortality, but not all-cause mortality. Therefore, some authors have a more critical point of view on the matter, suggesting that the results of randomized controlled trials, including the CONVINCE trial, and meta-analyses reporting a statistically significant reduction in the risk of all-cause mortality in patients undergoing online HDF compared to HD, were not very robust [55].

In the pediatric population, no RCTs have been performed to study the effects of HDF in contrast with HD [56]. However, the prospective multicenter 3H study, performed in 10 European countries, found an improvement in blood pressure control, reduced ventricular mass, with a slower increase in carotid intima-media thickness, and a reduction in inflammatory cytokines such as IL-6, TNF-α, and high sensitivity CRP in the HDF group [57,58]. In addition, fibroblast growth factor 23 decreased by 25% in the HDF group but increased in all patients on HD [58]. Quality of life was also enhanced, as there were fewer cramps, headaches, and lower post-dialysis recovery times, improving school attendance and physical activity [56]. Other studies have reported similar results, such as that by Fadel et al. [59], which found an improvement in systolic function in pediatric patients on HDF [59]. A study by Fischbach et al. [60] showed that HDF promoted growth in children receiving growth hormone treatment.

## 7. HDF’s Current Situation in Japan

Japanese HD population has a more extended period of chronic treatment and better clinical outcomes than American and European HD patients. This might be partly explained by the very low prevalence of renal transplantation in Japan. As a result, younger HD patients without comorbid severe conditions, whose prognosis should be good, have not been transplanted but have been treated by chronic HD therapy for a long period. Although online hemodiafiltration has a potential advantage over HF-HD, the impact of this therapy has not been evident because of its low prevalence in chronic dialysis therapy in Japan [61].

Postdilution HDF crucially depends on increasing the blood flow rate. Indeed, there is an important difference between the Qb used in Japan and Europe. In Europe, a Qb of 350 to 450 mL/min is commonly prescribed for more than 90% of patients, even those with tunneled catheters. Notably, when increasing the device’s blood flow rate, it is necessary to adapt the size of the metal needles or plastic cannulas [62].

According to the Japanese Society for Dialysis Therapy, 10.6% of HD treatments consist of HDF. Among those, 90.8% of patients who received online HDF used the predilution mode, and the mean substitution fluid volume per session was 40.6 L. On the other hand, only 9.2% of patients used the postdilution mode, with a mean volume of substitution fluid of 9.2 L [63]. One of the primary reasons for using the predilution mode for online HDF patients is the low blood flow rate, which averages 200 mL/min in Japanese patients. It is essential to understand that Qb is the main limiting factor of postdilutional HDF, as, to avoid hemoconcentration, it must be less than a third of it, usually between 25 and 30%. Predilutional HDF has no limitation on the replacement volume because it consists of a prefilter infusion. Additionally, it has a lower risk of coagulation of the extracorporeal circuit.

Predilution online HDF stabilizes circulatory dynamics and enables solute removal. It has high biocompatibility and can potentially improve pain management and nutrition status. It is also superior to postdilution online HDF in removing protein-binding uremic toxins (e.g., p-cresol and homocysteine). Thus, this treatment can potentially help overcome long-term complications in dialysis patients [64].

In addition, predilution HDF has also been associated with improved survival in Japan compared with hemodialysis [65]. Using data from the Japanese Society for Dialysis Therapy Renal Data Registry, a cohort of 5000 pairs of patients treated with either conventional hemodialysis or predilution online hemodiafiltration were analyzed. The study found that predilution online hemodiafiltration was associated with improved overall survival (HR 0.83) and a trend towards improved cardiovascular survival. This trend was statistically significant in patients treated with high substitution volumes (>40.0 L per session) compared to those treated with low substitution volumes (<40.0 L per session) or those on hemodialysis [65].

## 8. Expanded Hemodialysis (HDx) as an Alternative

Expanded HD (HDx) requires the use of a medium cutoff dialyzer and appears to offer a viable new alternative to hemodiafiltration (HDF). However, there are some questions about its efficacy compared to high-flux HD and HDF, especially in terms of dialyzer classification and clinical outcomes.

Dialyzer classification differs between Europe and Japan. In Europe, the classification includes low flux, high flux, medium cutoff (MCO), and high cutoff (HCO) based on specific coefficients and clearance rates. In contrast, Japan categorizes dialyzers based on their beta 2-microglobulin sieving coefficient, whereas those categorized as super high-flux or class V behave as MCO dialyzers [66]. In Japan, most patients (90%) undergoing hemodialysis are treated with super high-flux membranes. Of these dialyzers, 15% belong to type V, meaning they could be considered as MCO dialyzers [67]. A study conducted by Abe found an association between all-cause mortality and dialyzer type. The type V group had a significantly lower risk of 3-year mortality as compared to the reference type IV group [68]. Research comparing HDx to high-flux HD and HDF has shown intriguing insights. In a notable RCT involving 40 patients alternating between HF-HD and HDx for 3 months, each modality demonstrated promising results [69]. Moreover, the study highlighted the importance of lambda-free light chains as potential markers for assessing depurative efficacy within the molecular weight range of 40–45 KDa [69].

Comparative studies involving myoglobin, prolactin, and Kappa-free light chain patterns further emphasized the differences in depurative efficiency among HDx and other modalities [70]. Notably, despite differences in molecular weight removal rates, the Global Removal Score among MCO dialyzers was consistent, and it was significantly higher than with HD but lower than with postdilution HDF. Moreover, albumin dialysate loss remained clinically acceptable with these treatments [71]. 

Addressing safety concerns, long-term studies such as REMOVAL demonstrated the stability of pre-dialysis albumin levels over a 6-month follow-up, affirming the safety profile of HDx [72]. Similar conclusions were drawn in a Korean study with a 12-month follow-up period, supporting the safety and viability of HDx in clinical practice [73].

However, cautionary observations from case reports underscore the importance of judicious use of MCO, HDx, and super high-flux dialyzers, specifically within the HD modality. Instances of significant albumin loss and hypoalbuminemia during postdilution HDF stress the need for careful selection of patients and treatment modalities to prevent adverse outcomes [74].

Finally, while HDx shows promise as an alternative to HDF, this treatment modality lacks hard outcome data. An ongoing trial (NCT03714386) [75] is currently seeking to determine whether HDx is non-inferior to HDF and assess its role as an alternative in patients unable to achieve an adequate convective volume or in hemodialysis centers without access to an ultrapure water plant. Further studies will ascertain its efficacy, safety, and suitability for different patient profiles, ensuring optimal outcomes in renal replacement therapy.

## 9. Conclusions and Future Directions

The accumulating body of evidence overwhelmingly shows that HDF is a superior modality, demonstrating substantial clinical advantages and a clear impact on patient survival in comparison to high flux HD. This robust evidence provides strong support for the elevation of HDF to the status of the new ‘conventional’ hemodialysis technique, given its remarkable efficacy and proven benefits in enhancing patient outcomes.

However, amidst the remarkable success of HDF, the emergence of HDx offers an intriguing alternative, particularly for patients unable to receive more in-depth treatment with HDF. While initial studies suggest that HDx is a viable option, deeper investigation is required to define its precise role and suitability in diverse patient profiles. More extensive research is indispensable to consolidate its place as a genuine alternative, ensuring comprehensive treatment approaches for individuals undergoing dialysis.

## Figures and Tables

**Table 1:** Comparison of the different HDF randomized control studies.

	Italian Trial [23]	French Trial [22]	Contrast Study [37]	Turkish Study [38]	ESHOL [39]	Convince [40]
Country	Italian	French	Dutch	Turkish	Spain	Dutch
Included patients	146	420	714	782	906	1360
Year started	2004	2005	2004	2007	2007	2019
Year of publication	2008	2010	2010	2010	2011	2023
Compared groups	Pre-OL-HDF HF LF-HD 1:1:2	Post-OL-HDF HF-HD 1:1	Post-OL-HDF LF-HD 1:1	Post-OL-HDF HF-HD 1:1	Post-OL-HDF HF-HD 1:1	Post-OL-HDF HF-HD 1:1
Follow-up	2 years	2 years	3 years	2 years	3 years	2.5 years
Primary endpoints	Cardiovascular stability and BP control	Intradialytic tolerance	Mortality	Mortality and cardiovascular events	Mortality	Mortality
Results	No difference between groups	No difference between groups	HR 0.95 (0.83–1.39)	HR 0.82 (0.59–1.16)	HR 0.70 (0.53–0.92)	HR 0.77 (0.65–0.93)

BP: blood pressure; HF: hemofiltration; HF-HD: high-flux hemodialysis; HR: hazard ratio; LF-HD: low-flux hemodialysis; Post-OL-HDF: online postdilution hemodiafiltration; Pre-OL-HDF: online predilution hemodiafiltration.

**Table 2:** In-depth comparison of ESHOL and CONVINCE trials.

			ESHOL [39]		Convince [40]
Age (years)			65.4 ± 14.4		62.4 ± 13.5
Male sex			606 (66.9%)		856 (62.9%)
Type 2 diabetes			226 (24.9%)		481 (35.4%)
Did not complete treatment			355 (39.2%)		314 (23.1%)
Censored in analysis			Yes (Per protocol)		No (ITT)
Reasons					
Kidney transplantation			180 (19.6%)		146 (10.7%)
Change of HD center			58 (6.4%)		95 (7%)
Change of HD modality			48 (5.3%)		58 (4.3%)
Other			69 (7.9%)		15 (1.1%)
Dialysis vintage (months)			28 (12–59)		33 (15–72)
Tunneled catheters			93 (10.3%)		184 (13.5%)
Qb (mL/min)			387		368
Convective volume (L)			23.9		25.2
Time of treatment (min)			236		244
Inclusion period (months)			16		30
Study duration (months)			52		52
Mean follow-up (years)			1.91 ± 1.1		1.92 ± 1.1
Mortality (100 patients/year)			11.95		7.13
All-cause mortality					
Hazard ratio			0.70 (0.53–0.92)		0.77 (0.65–0.93)
NNT			10 (6–41)		22 (12–268)
All-cause mortality subgroup analysis					
	Age	Tertile 1	0.81 (0.36–1.81)	<50 yr	0.25 (0.06–1.05)
		Tertile 2	0.82 (0.51–1.33)	50 to 65 yr	1.05 (0.75–1.49)
		Tertile 3	0.63 (0.43–0.92)	>65 yr	0.68 (0.53–0.89)
	Diabetes	No	0.68 (0.48–0.95)	No	0.65 (0.48–0.87)
		Yes	0.75 (0.46–1.21)	Yes	0.97 (0.72–1.31)
	Vascular access	Fistula or graft Catheter	0.72 (0.53–0.97)	Fistula Graft or catheter	0.77 (0.64–0.94)
			0.83 (0.38–1.79)		0.78 (0.45–1.34)
Cardiovascular death			0.39 (0.16–0.93)		0.81 (0.49–1.33)
Other causes of death			0.45 (0.21–0.96)		0.76 (0.59–0.98)
Hospitalization			0.78 (0.67–0.90)		1.11 (0.98–1.25)

HD: hemodialysis; ITT: intention-to-treat; NNT: number needed to treat; Qb: blood flow rate; yr: year.

## Data Availability

Not applicable.

## References

[1] Henderson L.W., Beans E. (1978). Successful Production of Sterile Pyrogen-Free Electrolyte Solution by Ultrafiltration. Kidney Int..

[2] Canaud B., Flavier J.L., Argilés A., Stec F., Nguyen Q.V., Bouloux C., Garred L.J., Mion C. (1994). Hemodiafiltration with On-Line Production of Substitution Fluid: Long-Term Safety and Quantitative Assessment of Efficacy. Effective Hemodiafiltration: New Methods.

[3] Canaud B., Kerr P., Argiles A., Flavier J.L., Stec F., Mion C. (1993). Is Hemodiafiltration the Dialysis Modality of Choice for the next Decade?. Kidney Int. Suppl..

[4] Rocco M., Daugirdas J.T., Depner T.A., Inrig J., Mehrotra R., Rocco M.V., Suri R.S., Weiner D.E., Greer N., Ishani A. (2015). KDOQI Clinical Practice Guideline for Hemodialysis Adequacy: 2015 Update. Am. J. Kidney Dis..

[5] Maduell F. (2018). Hemodiafiltration versus Conventional Hemodialysis: Should “Conventional” Be Redefined?. Semin. Dial..

[6] Ronco C. (1990). Backfiltration in Clinical Dialysis: Nature of the Phenomenon, Mechanisms and Possible Solutions. Int. J. Artif. Organs.

[7] Tattersall J.E., Ward R.A., Canaud B., Blankestijn P.J., Bots M., Covic A., Davenport A., Grooteman M., Gura V., Hegbrant J. (2013). Online Haemodiafiltration: Definition, Dose Quantification and Safety Revisited. Nephrol. Dial. Transplant..

[8] Chapdelaine I., De Roij Van Zuijdewijn C.L.M., Mostovaya I.M., Lévesque R., Davenport A., Blankestijn P.J., Wanner C., Nubé M.J., Grooteman M.P.C. (2015). Optimization of the Convection Volume in Online Post-Dilution Haemodiafiltration: Practical and Technical Issues. Clin. Kidney J..

[9] Ahrenholz P., Winkler R.E., Ramlow W., Tiess M., Müller W. (1997). On-Line Hemodiafiltration with Pre- and Postdilution: A Comparison of Efficacy. Int. J. Artif. Organs.

[10] Maduell F., Ojeda R., Rodas L., Rico N., Fontsere N., Arias M., Vera M., Masso E., Jimenez-Hernandez M., Rossi M.F. (2015). On-Line Haemodiafiltration with Auto-Substitution: Assessment of Blood Flow Changes on Convective Volume and Efficiency. Nefrologia.

[11] Maduell F., Navarro V., Hernández-Jaras J., Calvo C. (2000). Comparison of Dialyzers in On-Line Hemodiafiltration. Nefrologia.

[12] Alvarez-de Lara M.A., Martín-Malo A. (2014). Hypersensitivity Reactions to Synthetic Haemodialysis Membranes—An Emerging Issue?. Nefrologia.

[13] Sunohara T., Masuda T. (2017). Fundamental Characteristics of the Newly Developed ATA^TM^ Membrane Dialyzer. Contrib. Nephrol..

[14] Maduell F., Ojeda R., Arias-Guillén M., Fontseré N., Vera M., Rodas L., Gómez M., Huablocho K.P., Esquivel F., Mori P.D. (2018). A New Generation of Cellulose Triacetate Suitable for Online Haemodiafiltration. Nefrologia.

[15] Oshihara W., Fujieda H., Ueno Y. (2017). A New Poly(Methyl Methacrylate) Membrane Dialyzer, NF, with Adsorptive and Antithrombotic Properties. Contrib. Nephrol..

[16] Maduell F., Broseta J.J., Rodríguez-Espinosa D., Hermida-Lama E., Rodas L.M., Gómez M., Arias-Guillén M., Fontseré N., Vera M., Rico N. (2021). Evaluation and Comparison of Polysulfone TS-UL and PMMA NF-U Dialyzers versus Expanded Hemodialysis and Postdilution Hemodiafiltration. Artif. Organs.

[17] Maduell F., Ojeda R., Belmar L., Munguía P., Sango C., Martinez-Díaz A.I., Arias-Guillén M., Vera M., Fontseré N., Gómez M. (2018). Evaluation of the Dialyzer Inner Diameter in Online Haemodiafiltration. Nefrología Engl. Ed..

[18] Penne E.L., van der Weerd N.C., van den Dorpel M.A., Grooteman M.P.C., Lévesque R., Nubé M.J., Bots M.L., Blankestijn P.J., ter Wee P.M. (2010). Short-Term Effects of Online Hemodiafiltration on Phosphate Control: A Result From the Randomized Controlled Convective Transport Study (CONTRAST). Am. J. Kidney Dis..

[19] Rama I., Llaudó I., Fontova P., Cerezo G., Soto C., Javierre C., Hueso M., Montero N., Martínez-Castelao A., Torras J. (2016). Online Haemodiafiltration Improves Inflammatory State in Dialysis Patients: A Longitudinal Study. PLoS ONE.

[20] Panichi V., Scatena A., Rosati A., Giusti R., Ferro G., Malagnino E., Capitanini A., Piluso A., Conti P., Bernabini G. (2015). High-Volume Online Haemodiafiltration Improves Erythropoiesis-Stimulating Agent (ESA) Resistance in Comparison with Low-Flux Bicarbonate Dialysis: Results of the REDERT Study. Nephrol. Dial. Transplant..

[21] Marcelli D., Bayh I., Merello J.I., Ponce P., Heaton A., Kircelli F., Chazot C., Di Benedetto A., Marelli C., Ladanyi E. (2016). Dynamics of the Erythropoiesis Stimulating Agent Resistance Index in Incident Hemodiafiltration and High-Flux Hemodialysis Patients. Kidney Int..

[22] Morena M., Jaussent A., Chalabi L., Leray-Moragues H., Chenine L., Debure A., Thibaudin D., Azzouz L., Patrier L., Maurice F. (2017). Treatment Tolerance and Patient-Reported Outcomes Favor Online Hemodiafiltration Compared to High-Flux Hemodialysis in the Elderly. Kidney Int..

[23] Locatelli F., Altieri P., Andrulli S., Bolasco P., Sau G., Pedrini L.A., Basile C., David S., Feriani M., Montagna G. (2010). Hemofiltration and Hemodiafiltration Reduce Intradialytic Hypotension in ESRD. J. Am. Soc. Nephrol..

[24] Mion M., Kenrr P.G., Argiles A., Canaud B., Flavier J.L., Mion C.M. (1992). Haemodiafiltration in High-Cardiovascular-Risk Patients. Nephrol. Dial. Transplant..

[25] Ohtake T., Oka M., Ishioka K., Honda K., Mochida Y., Maesato K., Moriya H., Hidaka S., Kobayashi S. (2012). Cardiovascular Protective Effects of On-Line Hemodiafiltration: Comparison With Conventional Hemodialysis. Ther. Apher. Dial..

[26] Bellien J., Fréguin-Bouilland C., Joannidès R., Hanoy M., Rémy-Jouet I., Monteil C., Iacob M., Martin L., Renet S., Vendeville C. (2014). High-Efficiency on-Line Haemodiafiltration Improves Conduit Artery Endothelial Function Compared with High-Flux Haemodialysis in End-Stage Renal Disease Patients. Nephrol. Dial. Transplant..

[27] Dekker M., Pasch A., Dersande F., Konings C., Bachtler M., Dionisi M., Meier M., Kooman J., Canaud B. (2016). High-Flux Hemodialysis and High-Volume Hemodiafiltration Improve Serum Calcification Propensity. PLoS ONE.

[28] Shinzato T., Maeda K. (2007). Push/Pull Hemodiafiltration. Contrib. Nephrol..

[29] Maeda K., Kobayakawa H., Fujita Y., Takai I., Morita H., Emoto Y., Miyazaki T., Shinzato T. (1990). Effectiveness of Push/Pull Hemodiafiltration Using Large-Pore Membrane for Shoulder Joint Pain in Long-Term Dialysis Patients. Artif. Organs.

[30] Rodríguez-Ribera L., Pastor S., Corredor Z., Silva I., Diaz J.M., Ballarin J., Marcos R., Coll E. (2016). Genetic Damage in Patients Moving from Hemodialysis to Online Hemodiafiltration. Mutagenesis.

[31] Karkar A., Abdelrahman M., Locatelli F. (2015). A Randomized Trial on Health-Related Patient Satisfaction Level with High-Efficiency Online Hemodiafiltration versus High-Flux Dialysis. Blood Purif..

[32] Vilar E., Fry A.C., Wellsted D., Tattersall J.E., Greenwood R.N., Farrington K. (2009). Long-Term Outcomes in Online Hemodiafiltration and High-Flux Hemodialysis: A Comparative Analysis. Clin. J. Am. Soc. Nephrol..

[33] Panichi V., Rizza G.M., Paoletti S., Bigazzi R., Aloisi M., Barsotti G., Rindi P., Donati G., Antonelli A., Panicucci E. (2008). Chronic Inflammation and Mortality in Haemodialysis: Effect of Different Renal Replacement Therapies. Results from the RISCAVID Study. Nephrol. Dial. Transplant..

[34] Jirka T., Cesare S., Di Benedetto A., Chang M.P., Ponce P., Richards N., Tetta C., Vaslaky L. (2006). Mortality Risk for Patients Receiving Hemodiafiltration versus Hemodialysis. Kidney Int..

[35] Canaud B., Bragg-Gresham J.L., Marshall M.R., Desmeules S., Gillespie B.W., Depner T., Klassen P., Port F.K. (2006). Mortality Risk for Patients Receiving Hemodiafiltration versus Hemodialysis: European Results from the DOPPS. Kidney Int..

[36] Vinhas J., Vaz Á., Barret C., Assunção J. (2007). Survival Advantage of Patients on Haemodiafiltration Is Independent of Dialysis Dose and Patient Characteristics: Data from a Single Centre. Port. J. Nephrol. Hypertens..

[37] Penne E.L., Blankestijn P.J., Bots M.L., van den Dorpel M.A., Grooteman M.P., Nubé M.J., van der Tweel I., ter Wee P.M. (2005). Effect of Increased Convective Clearance by On-Line Hemodiafiltration on All Cause and Cardiovascular Mortality in Chronic Hemodialysis Patients—The Dutch Convective Transport Study (CONTRAST): Rationale and Design of a Randomised Controlled Trial [ISRCTN38365125]. Curr. Control. Trials Cardiovasc. Med..

[38] Ok E., Asci G., Toz H., Ok E.S., Kircelli F., Yilmaz M., Hur E., Demirci M.S., Demirci C., Duman S. (2013). Mortality and Cardiovascular Events in Online Haemodiafiltration (OL-HDF) Compared with High-Flux Dialysis: Results from the Turkish OL-HDF Study. Nephrol. Dial. Transplant..

[39] Maduell F., Moreso F., Pons M., Ramos R., Mora-Macià J., Carreras J., Soler J., Torres F., Campistol J.M., Martinez-Castelao A. (2013). High-Efficiency Postdilution Online Hemodiafiltration Reduces All-Cause Mortality in Hemodialysis Patients. J. Am. Soc. Nephrol..

[40] Blankestijn P.J., Vernooij R.W.M., Hockham C., Strippoli G.F.M., Canaud B., Hegbrant J., Barth C., Covic A., Cromm K., Cucui A. (2023). Effect of Hemodiafiltration or Hemodialysis on Mortality in Kidney Failure. N. Engl. J. Med..

[41] Mostovaya I.M., Blankestijn P.J., Bots M.L., Covic A., Davenport A., Grooteman M.P.C., Hegbrant J., Locatelli F., Vanholder R., Nubé M.J. (2014). Clinical Evidence on Hemodiafiltration: A Systematic Review and a Meta-Analysis. Semin. Dial..

[42] Mercadal L., Franck J.E., Metzger M., Urena Torres P., de Cornelissen F., Edet S., Béchade C., Vigneau C., Drüeke T., Jacquelinet C. (2016). Hemodiafiltration Versus Hemodialysis and Survival in Patients With ESRD: The French Renal Epidemiology and Information Network (REIN) Registry. Am. J. Kidney Dis..

[43] See E.J., Hedley J., Agar J.W.M., Hawley C.M., Johnson D.W., Kelly P.J., Lee V.W., Mac K., Polkinghorne K.R., Rabindranath K.S. (2019). Patient Survival on Haemodiafiltration and Haemodialysis: A Cohort Study Using the Australia and New Zealand Dialysis and Transplant Registry. Nephrol. Dial. Transplant..

[44] Marinovich S., Bisigniano L., Hansen Krogh D., Celia E., Tagliafichi V., Rosa Diez G., Fayad A. (2019). Registro Argentino de Diálisis Crónica SAN-INCUCAI 2019.

[45] da Rocha E.P., Kojima C.A., Modelli de Andrade L.G., Costa D.M., Magalhaes A.O., Rocha W.F., de Vasconcelos Junior L.N., Rosa M.G., Wagner Martins C.S. (2024). Comparing Survival Outcomes between Hemodialysis and Hemodiafiltration Using Real-World Data from Brazil. J. Clin. Med..

[46] Barrera L.A., Cantor E.J., Muñoz J., Arango J., Valderrama L.A., Barrera L., Cantor E.J., Muñoz J., Arango J., Tobon C. (2022). Mortality in High-Flux Hemodialysis vs. High-Volume Hemodiafiltration in Colombian Clinical Practice: A Propensity Score Matching Study. Kidney Dial..

[47] Neri L., Gurevich K., Zarya Y., Plavinskii S., Bellocchio F., Stuard S., Barbieri C., Canaud B. (2021). Practice Patterns and Outcomes of Online Hemodiafiltration: A Real-World Evidence Study in a Russian Dialysis Network. Blood Purif..

[48] Davenport A., Peters S.A.E., Bots M.L., Canaud B., Grooteman M.P.C., Asci G., Locatelli F., Maduell F., Morena M., Nubé M.J. (2016). Higher Convection Volume Exchange with Online Hemodiafiltration Is Associated with Survival Advantage for Dialysis Patients: The Effect of Adjustment for Body Size. Kidney Int..

[49] Canaud B., Barbieri C., Marcelli D., Bellocchio F., Bowry S., Mari F., Amato C., Gatti E. (2015). Optimal Convection Volume for Improving Patient Outcomes in an International Incident Dialysis Cohort Treated with Online Hemodiafiltration. Kidney Int..

[50] Astley M.E., Boenink R., Abd ElHafeez S., Trujillo-Alemán S., Arribas F., Åsberg A., Beckerman P., Bell S., Bouzas-Caamaño M.E., Farnés J.C. (2023). The ERA Registry Annual Report 2020: A Summary. Clin. Kidney J..

[51] Maduell F., Moreso F., Pons M., Ramos R., Mora-Maciá J., Carreras J., Soler J., Torres F., Campistol J.M., Martinez-Castelao A. (2014). Erratum: High-Efficiency Postdilution Online Hemodiafiltration Reduces All-Cause Mortality in Hemodialysis Patients (Journal of the American Society of Nephrology (2013) 24 (487–497)). J. Am. Soc. Nephrol..

[52] Caskey F.J., Procter S., MacNeill S.J., Wade J., Taylor J., Rooshenas L., Liu Y., Annaw A., Alloway K., Davenport A. (2022). The High-Volume Haemodiafiltration vs High-Flux Haemodialysis Registry Trial (H4RT): A Multi-Centre, Unblinded, Randomised, Parallel-Group, Superiority Study to Compare the Effectiveness and Cost-Effectiveness of High-Volume Haemodiafiltration and High-Flux Haemodialysis in People with Kidney Failure on Maintenance Dialysis Using Linkage to Routine Healthcare Databases for Outcomes. Trials.

[53] Locatelli F., Karaboyas A., Pisoni R.L., Robinson B.M., Fort J., Vanholder R., Rayner H.C., Kleophas W., Jacobson S.H., Combe C. (2018). Mortality Risk in Patients on Hemodiafiltration versus Hemodialysis: A “real-World” Comparison from the DOPPS. Nephrol. Dial. Transplant..

[54] Nistor I., Palmer S.C., Craig J.C., Saglimbene V., Vecchio M., Covic A., Strippoli G.F.M. (2015). Haemodiafiltration, Haemofiltration and Haemodialysis for End-Stage Kidney Disease. Cochrane Database Syst. Rev..

[55] Battaglia Y., Mantovani A., Shroff R., Alfano G., Meijers B., Franssen C., Combe C., Basile C. (2024). Online Haemodiafiltration and All-Cause Mortality: How Fragile Are the Results of the Studies Published so Far?. Nephrol. Dial. Transplant..

[56] Shroff R., Smith C., Ranchin B., Bayazit A.K., Stefanidis C.J., Askiti V., Azukaitis K., Canpolat N., Agba A., Aitkenhead H. (2019). Effects of Hemodiafiltration versus Conventional Hemodialysis in Children with ESKD: The HDF, Heart and Height Study. J. Am. Soc. Nephrol..

[57] Ağbaş A., Canpolat N., Çalışkan S., Yılmaz A., Ekmekçi H., Mayes M., Aitkenhead H., Schaefer F., Sever L., Shroff R. (2018). Hemodiafiltration Is Associated with Reduced Inflammation, Oxidative Stress and Improved Endothelial Risk Profile Compared to High-Flux Hemodialysis in Children. PLoS ONE.

[58] Fischer D.C., Smith C., De Zan F., Bacchetta J., Bakkaloglu S.A., Agbas A., Anarat A., Aoun B., Askiti V., Azukaitis K. (2021). Hemodiafiltration Is Associated With Reduced Inflammation and Increased Bone Formation Compared With Conventional Hemodialysis in Children: The HDF, Hearts and Heights (3H) Study. Kidney Int. Rep..

[59] Fadel F.I., Makar S.H., Zekri H., Ahmed D.H., Aon A.H. (2015). The Effect of On-Line Hemodiafiltration on Improving the Cardiovascular Function Parameters in Children on Regular Dialysis. Saudi J. Kidney Dis. Transplant..

[60] Fischbach M., Terzic J., Menouer S., Dheu C., Seuge L., Zalosczic A. (2010). Daily on Line Haemodiafiltration Promotes Catch-up Growth in Children on Chronic Dialysis. Nephrol. Dial. Transplant..

[61] Shinoda T., Koda Y. (2011). Clinical Evaluation Indices for Hemodialysis/Hemodiafiltration in Japan. Contrib. Nephrol..

[62] Cho A., Park H.C., Kim D.H., Choi H.B., Song G.H., Kim H., Kim S.H., Choi G., Kim J.K., Song Y.R. (2023). Impact of Needle Type on Substitution Volume during Online Hemodiafiltration: Plastic Cannulae versus Metal Needles. Kidney Res. Clin. Pract..

[63] Masakane I., Nakai S., Ogata S., Kimata N., Hanafusa N., Hamano T., Wakai K., Wada A., Nitta K. (2015). An Overview of Regular Dialysis Treatment in Japan (As of 31 December 2013). Ther. Apher. Dial..

[64] Tsuchida K., Minakuchi J. (2013). Clinical Benefits of Predilution On-Line Hemodiafiltration. Blood Purif..

[65] Kikuchi K., Hamano T., Wada A., Nakai S., Masakane I. (2019). Predilution Online Hemodiafiltration Is Associated with Improved Survival Compared with Hemodialysis. Kidney Int..

[66] García-Prieto A., de la Flor J.C., Coll E., Iglesias E., Reque J., Valga F. (2023). Expanded Hemodialysis: What’s up, Doc?. Clin. Kidney J..

[67] Abe M., Hamano T., Wada A., Nakai S., Masakane I. (2017). High-Performance Membrane Dialyzers and Mortality in Hemodialysis Patients: A 2-Year Cohort Study from the Annual Survey of the Japanese Renal Data Registry. Am. J. Nephrol..

[68] Abe M., Masakane I., Wada A., Nakai S., Nitta K., Nakamoto H. (2021). Super High-Flux Membrane Dialyzers Improve Mortality in Patients on Hemodialysis: A 3-Year Nationwide Cohort Study. Clin. Kidney J..

[69] Belmouaz M., Bauwens M., Hauet T., Bossard V., Jamet P., Joly F., Chikhi E., Joffrion S., Gand E., Bridoux F. (2020). Comparison of the Removal of Uraemic Toxins with Medium Cut-off and High-Flux Dialysers: A Randomized Clinical Trial. Nephrol. Dial. Transplant..

[70] Kim T.H., Kim S.H., Kim T.Y., Park H.Y., Jung K.S., Lee M.H., Jhee J.H., Lee J.E., Choi H.Y., Park H.C. (2019). Removal of Large Middle Molecules via Haemodialysis with Medium Cut-off Membranes at Lower Blood Flow Rates: An Observational Prospective Study. BMC Nephrol..

[71] Maduell F., Broseta J.J., Rodríguez-Espinosa D., Del Risco J., Rodas L.M., Arias-Guillén M., Vera M., Fontseré N., Salgado M.D.C., Rico N. (2022). Comparison of Four Medium Cut-off Dialyzers. Clin. Kidney J..

[72] Krishnasamy R., Hawley C.M., Jardine M.J., Roberts M.A., Cho Y., Wong M., Heath A., Nelson C.L., Sen S., Mount P.F. (2020). A TRial Evaluating Mid Cut-Off Value Membrane Clearance of Albumin and Light Chains in HemoDialysis Patients: A Safety Device Study. Blood Purif..

[73] Cho N.J., Park S., Islam M.I., Song H.Y., Lee E.Y., Gil H.W. (2019). Long-Term Effect of Medium Cut-off Dialyzer on Middle Uremic Toxins and Cell-Free Hemoglobin. PLoS ONE.

[74] Cuvelier C., Tintillier M., Migali G., Van Ende C., Pochet J.M. (2019). Albumin Losses during Hemodiafiltration: All Dialyzers Are Not Created Equal—A Case Report. BMC Nephrol..

[75] de Sequera P., Pérez-García R., Vega A., Martínez-Vaquera S., Acosta J.G., Pérez Del Valle K., Fernández-Lucas M., García-Rubiales M.A., García-Herrera A.L., Coll E. (2023). Trial Design of the MOTheR HDx Study: A Multicenter, Open-Label, Prospective, Randomized Study to Explore the Morbidity and Mortality in Patients Dialyzed with the Theranova HDx in Comparison with Online Hemodiafiltration. Clin. Kidney J..

